# External validation of machine learning models for estimation of mortality 1, 3, 6, and 12 months after hip fracture on 5,055 consecutive patients

**DOI:** 10.2340/17453674.2026.45871

**Published:** 2026-06-10

**Authors:** Mathias MOSFELDT, Henrik L JØRGENSEN, Jes B LAURITZEN, Karl-Åke JANSSON

**Affiliations:** 1 Department of Orthopaedics, Karolinska University Hospital, Stockholm; 2 Department of Molecular Medicine and Surgery, Karolinska Institutet, Stockholm, Sweden; 3 Department of Clinical Biochemistry, Hvidovre Hospital, University of Copenhagen; 4 Department of Clinical Medicine, University of Copenhagen, Copenhagen; 5 Department of Orthopaedic Surgery, Bispebjerg Hospital, University of Copenhagen, Copenhagen, Denmark; 6 Department of Clinical Science and Education Södersjukhuset, Karolinska Institutet, Unit of Orthopaedics, Södersjukhuset, Sweden

## Abstract

**Background and purpose:**

Many models for prediction of mortality after hip fracture have been published but few have undergone external validation, usually considered a prerequisite for assessing the actual precision before being put to clinical use. The aim of our study was to externally validate our previously published models in an independent Swedish cohort.

**Methods:**

We retrospectively analyzed 5,055 consecutive patients with hip fractures from 2 hospitals in Stockholm, Sweden, between 2010 and 2020. Previously developed Random Forest (RF), eXtreme Gradient Boosting (XGB), and Generalized Linear Models (GLM) (Mosfeldt et al. 2024) were deployed to estimate mortality at 1, 3, 6, and 12 months. Model performance was assessed using the area under the receiver operating characteristic curve (AUC), calibration metrics, and decision curve analysis (DCA). Bootstrapped isotonic regression was used for recalibration.

**Results:**

All models showed acceptable performance, with XGB performing best (AUC 0.72, 0.74, 0.75, and 0.77 for 1-, 3-, 6-, and 12-month mortality). Mortality was lower in the validation cohort, so the models were recalibrated to adjust for this difference.

**Conclusion:**

External validation of the previously published models confirmed the original findings, with the XGB models again demonstrating the best overall performance. Recalibration addressed cohort differences in mortality rates and resulted in well-aligned predictions. The updated models for 3- and 12-month mortality are available online (https://hipfx.shinyapps.io/hipfxswe/), allowing clinicians to input patient data and receive individualized mortality predictions to support clinical decision-making.

Fragility hip fractures are associated with excess mortality and substantial healthcare costs that are projected to increase [1]. Accurate estimation of post-fracture mortality can guide clinical decision-making and help target care and research efforts to where they are most needed. While traditional prognostic models exist, machine learning (ML) offers opportunities for more precise and personalized risk prediction. Several ML models have been proposed to estimate mortality after hip fracture [2,3], but few have undergone external validation. External validation is required to assess model performance across patient cohorts and healthcare settings before clinical use. Differences in mortality rates and clinical variables may require recalibration or updating when models are applied to new populations. In this study, we externally validate our previously published ML models [4] for predicting mortality after hip fracture in a large, independent cohort.

## Methods

### Source of data

The patients for the study came from 2 university hospitals in Stockholm and 2 separate registries were merged to get the necessary parameters to externally validate the models that were developed in our previous study [4].

The Swedish National Hip Fracture Registry (RIKSHÖFT) is a national registry for all hip fracture patients that collect a wide range of data. From this registry we were able to collect data on mobilization and walking aids, permanent living arrangements, where patients were admitted from, admission date, age, body mass index (BMI), sex, American Society of Anesthesiologists Physical Status classification (ASA) grade, and time of death if this had occurred.

From the Karolinska University Hospital database (KARDA) we extracted data concerning admission blood sample levels (creatinine, potassium, albumin, hemoglobin, calcium) and medication (cardiac medication: yes/no, any of the following alone or in any combination: diuretics, betablockers, digoxin, vitamin K antagonists, organic nitrates). Only preoperative blood samples were used. Data on all patients who were admitted with a hip fracture between January 1, 2010 and December 31, 2020 was collected.

Data on mortality has been collected from the Swedish civil registration system where all persons legally residing permanently in Sweden are registered. Mortality data was collected through December 31, 2021, ensuring at least 1 year of follow-up for all patients.

The manuscript was prepared according to the TRIPOD guidelines for Transparent Reporting of a Multivariable Prediction Model for Individual Prognosis or Diagnosis [5].

### Participants

The 2 hospitals in the study, Karolinska University Hospital, Huddinge and Solna, are individual hospitals situated in different parts of Stockholm but under the same general administration. Both are tertiary care centers.

All patients above the age of 60 years without known pathological or metastatic fractures who were admitted during the study period were included. Patients who were registered as ASA 5 were excluded.

All patients were treated operatively and according to local guidelines that include a fast-track program and the use of femoralis or fascia iliaca blocks in order to decrease the use of opiates. Furthermore, a local guideline for choosing operation method inspired by Palm et al. [6] was in place for most of the study period.

### Outcome

The outcome predicted is likelihood of not surviving 1, 3, 6, and 12 months after sustaining a hip fracture.

The article describing the development of these models was published in 2024 [4].

### Predictors

The blood samples in the study were converted to categories according to reference intervals in the study population. BMI, age, sex, and ASA score were recorded in a similar manner in both original development of the models and in the data for the external validation. Presence of any “cardiac medication” in the patient’s regular medication recorded on admission was categorized. The factors “Permanent living arrangements” and “Admitted from” had similar categories in both datasets so they were simply translated.

Finally, the parameter “New Mobility Score” (NMS) [7] that was used in the development of the models was not recorded in the datasets for the evaluation. In the data for external validation, 2 categories existed with 5 levels each. These described levels of mobilization from uninhibited to completely dependent/bedridden and the use of walking aids from no aids to wheelchair. Combining the 2 resulted in a scale of 2–10 from fully independent and using no aids to bedridden/wheelchair with no ability to walk. In effect, the individual levels were not necessarily worded in an identical manner as in the NMS, but both were scales of independence in walking and need for walking aids and was therefore interpreted as having a similar effect. As there was a different number of levels in the 2, different combinations of levels in the external validation data were translated to create an extra level to match the development data.

Walking independently indoors with a walker was valued higher than walking only with company indoors and 2 crutches, creating levels 4 and 3 in the NMS respectively.

### Missing data

Missing data was handled with multiple imputation using the same random forest imputation method [8] as in the original study. There were 8,671 missing values out of 85,935 values.

### Statistics

Predictions were calculated retrospectively using the same models that were developed in the original study after the parameters had been categorized accordingly and given matching labels.

Model performance was evaluated using discrimination (area under the ROC curve [AUC]) with 95% confidence intervals (CI), calibration (calibration intercept, slope, and flexible calibration curves), and clinical usefulness using decision curve analysis (DCA), as suggested by Steyerberg et al. [9]. While discrimination is summarized by a single parameter and therefore reported with confidence intervals, calibration and DCA are primarily evaluated through intercept/slope estimates and graphical assessment of calibration curves and net benefit across threshold probabilities, respectively, for which a single standard confidence interval is not typically reported.

AUC measures the ability of the model to separate patients who die at a given time interval from those who survive. Higher AUC values indicate better discrimination. While there are no universally accepted thresholds, convention often considers values above 0.7 as acceptable, above 0.8 as good, and above 0.9 as excellent discrimination.

Calibration was evaluated using intercept, slope, and flexible calibration curves, which show agreement between predicted and observed probabilities. An intercept near 0 and slope near 1 indicate good calibration. Flexible calibration curves highlight areas of systematic over- or underestimation; perfect calibration is represented by the diagonal 45° line. Calibration uncertainty was assessed using intercept and slope.

DCA evaluates the clinical usefulness of the model by quantifying net benefit across a range of threshold probabilities for intervention. A model with higher net benefit than “treat all” or “treat none” strategies across relevant thresholds is considered clinically useful [10].

It was noted that the XGB model had acceptable discrimination but was consistently overestimating mortality in line with the observation that mortality rates were higher in the development set, indicating that recalibration for the population under study should be performed. Recalibration adjusts the mapping between model scores and predicted probabilities to reflect the event rate in the validation population, while preserving the ranking of predictions and therefore the AUC. The AUC and its associated uncertainty therefore remain unchanged, while the predicted probabilities become better aligned with the observed outcomes [11,12].

As the calibration curves were monotonic and non-sigmoidal, a bootstrapped isotonic regression was chosen in favor of a Platt-scaling/logistic regression approach.

The recalibrated models had an average predicted risk much closer to the event rate at all timepoints and the improved calibration curves for 3- and 12-month mortality models are presented in Figure 4, and metrics for all models in Table 2.

Rstudio, version 2023.03.0+386 (R Foundation for Statistical Computing, Vienna, Austria) was used for calculations.

### Ethics, funding, and disclosures

The study was approved by the Swedish Ethical Review Authority (reference number 2021-05298). No external funding was received for this study. The authors declare no competing interests. Complete disclosure of interest forms according to ICMJE are available on the article page, doi: 10.2340/17453674.2026.45871

## Results

**Figure 1:**
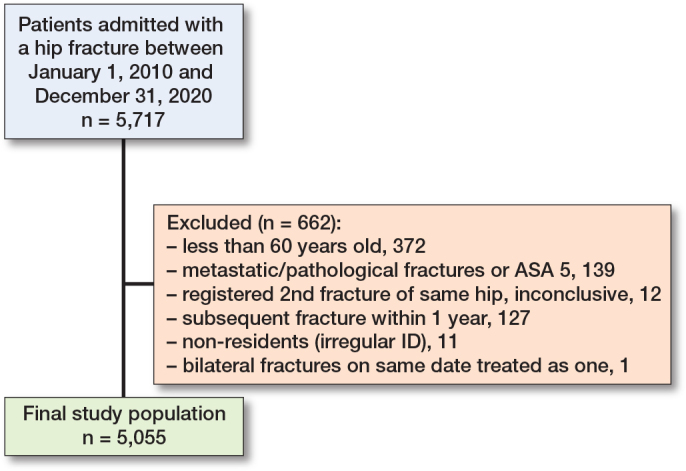
Flowchart of patient selection outlines the exclusion process and how the final dataset of patients was derived from the initial cohort.

The dataset included 5,717 patients. Second fractures within 1 year were treated as new events (n = 127), censoring the first fracture, while fractures occurring after 1 year were treated as separate events (n = 187). Each event initiated a 12-month mortality follow-up [13]. This left 5,055 patients after exclusions; details are given in a flowchart (Figure 1). Participants were characterized using categorical variables reported as n (%) and continuous variables reported as mean (SD), stratified by 1-year survival, with corresponding development data presented for comparison (Table 1 and 2). No data was missing for the outcome and approximately 10% was missing for the predictors.

**Table 1:** Descriptive baseline characteristics stratified by mortality and comparisons with the development data. Values are mean (standard deviation) unless otherwise specified

Factor	External validation cohort stratified by 1-year survival		Development cohort stratified by 1-year survival	
	No	Yes	No	Yes
Survival 1 year, n (%)	1,376 (27)	3,679 (73)	378 (32)	808 (68)
Age	85.2 (8.2)	81.0 (8.9)	86.1 (8.4)	81.3 (9.2)
Male sex, n (%)	541 (39)	1,106 (30)	104 (28)	202 (25)
Body mass index	22.9 (3.0)	23.9 (2.9)	22.1 (3.5)	22.9 (4.2)
Creatinine, μmol/L	104.4 (78.6)	85.5 (54.5)	103.8 (74.1)	77.7 (36.5)
Hemoglobin, g/L	119.1 (17.8)	125.7 (16.2)	–	–
Hemoglobin, mmol/L	–	–	7.36 (1.03)	7.74 (1.05)
Potassium, mmol/L	4.18 (0.54)	4.07 (0.51)	4.02 (0.60)	3.86 (0.48)
Calcium, mmol/L	2.25 (0.14)	2.26 (0.12)	2.26 (0.17)	2.26 (0.13)
Albumin, g/L	31.6 (5.0)	34.5 (4.2)	36.5 (5.3)	38.7 (4.3)
Admitted from, n (%)				
Assisted living	86 (6.2)	154 (4.2)	18 (4.8)	52 (6.4)
Hospice	1 (0.1)	4 (0.1)	2 (0.5)	0 (0.0)
Hospital	72 (5.2)	143 (3.9)	7 (1.9)	11 (1.4)
Nursing home	488 (35)	606 (16)	155 (41)	126 (16)
Own home	681 (50)	2,720 (74)	180 (48)	603 (75)
Rehabilitation center	48 (3.5)	52 (1.4)	16 (4.2)	16 (2.0)
Permanent residence, n (%)				
Nursing home	651 (47)	882 (24)	154 (41)	129 (16)
Own home	725 (53)	2,797 (76)	220 (58)	675 (84)
New Mobility Score, n (%)				
0	107 (7.8)	100 (2.7)	15 (4.0)	10 (1.2)
1	18 (1.3)	35 (1.0)	8 (2.1)	6 (0.7)
2	112 (8.1)	131 (3.6)	60 (16)	67 (8.3)
3	1 (0.1)	1 (0.0)	29 (7.7)	49 (6.1)
4	461 (34)	613 (17)	60 (16)	83 (10)
5	96 (7.0)	191 (5.2)	12 (3.2)	46 (5.7)
6	218 (16)	575 (16)	54 (14)	116 (14)
7	147 (11)	348 (9.5)	11 (2.9)	50 (6.2)
8	70 (5.1)	297 (8.1)	1 (0.3)	15 (1.9)
9	146 (11)	1,388 (38)	83 (22)	315 (39)
ASA classification, n (%)				
1	2 (0.1)	87 (2.4)	7 (1.9)	68 (8.4)
2	123 (8.9)	1,040 (28)	134 (35)	435 (54)
3	974 (71)	2,271 (62)	194 (52)	266 (33)
4	277 (20)	281 (7.6)	20 (5.3)	15 (1.9)
Cardiac medication, n (%)	614 (45)	1,289 (35)	236 (62)	358 (44)

**Table 2:** Mortality in validation and development cohorts. Values count (%)

Mortality	External validation cohort n = 5,055	Development cohort n = 1,186
1 month	425 (8.4)	145 (12)
3 months	756 (15)	242 (20)
6 months	1,015 (20)	308 (26)
1 year	1,376 (27)	378 (32)

Mortality across all timepoints was lower in the external validation cohort compared with the original development cohort (27% vs 32% at 1 year). Mean age was comparable (82 vs 83 years), but there were differences in sex distribution, ASA classification, cardiac medication use, and admission source, with the validation cohort including more males, more patients with higher ASA grades, greater cardiac medication use, and a higher proportion admitted from nursing homes. In accordance with the results from the original development of the models, the best performance overall was from the XGB model, with the exception of the AUC for 1-month mortality.

The XGB model had AUC values of 0.72 (CI 0.70–0.74), 0.74 (CI 0.72–0.76), 0.75 (CI 0.73–0.76), and 0.77 (CI 0.75–0.78) for 1-, 3-, 6-, and 12-month mortality respectively, while the RF model had slightly lower scores except for the 1-month model that had an AUC of 0.74.

The calibration at large and the flexible calibration curves were initially slightly off but were greatly improved by recalibration with bootstrapped isotonic regression. While the actual overall event rate in the test set was 0.08, 0.15, 0.20, and 0.27 for 1-, 3-, 6-, and 12-month mortality respectively, the average predicted risk using the XGB model was 0.12, 0.22, 0.30, and 0.38 for the same timepoints. After recalibration the same predicted risk was 0.07, 0.12, 0.17, amd 0.24 respectively and showed minor underestimation but much improved results.

The DCA showed net benefit across a wide range of thresholds, indicating the model’s usefulness in a clinical setting. Thresholds were set wider as the intended use of the model is not necessarily for a specific clinical decision and in some instances could simply be informative. As the results for the 1-month model were less accurate and as it was deemed unnecessary to have estimations for all the time points, results for 3- and 12-month mortality are reported. These recalibrated models have been incorporated into an updated online calculator (https://hipfx.shinyapps.io/hipfxswe/), enabling clinicians to apply the models directly in practice by entering patient-specific data to obtain individualized risk predictions.

**Figure 2:**
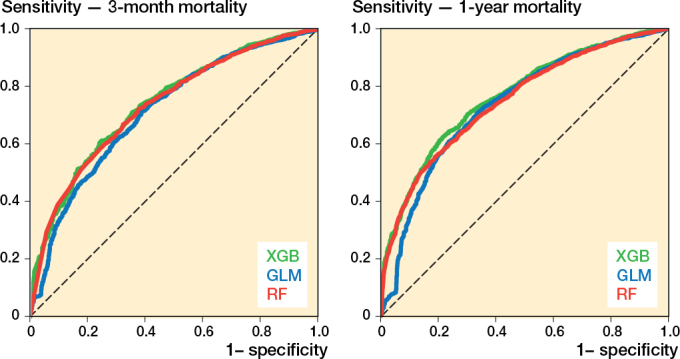
ROC curves and AUC for external validation of XGB, GLM, and RF models for 3-months and 1-year mortality after hip fracture.

**Figure 3:**
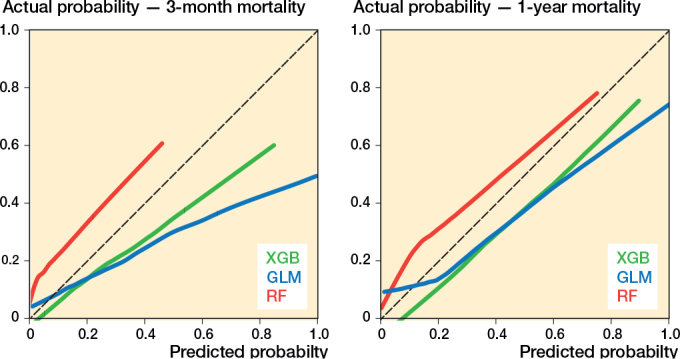
Flexible calibration curves for external validation of GLM, XGBoost, and RF models for 3-months and 1-year mortality.

**Figure 4:**
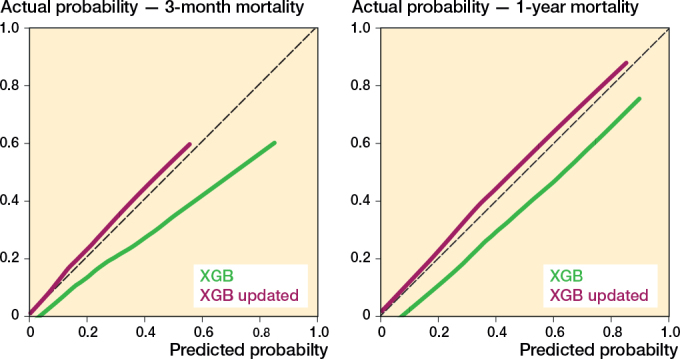
Flexible calibration curves for external validation of XGBoost model for 3-months and 1-year mortality, before (green) and after (purple) re-calibration.

**Figure 5:**
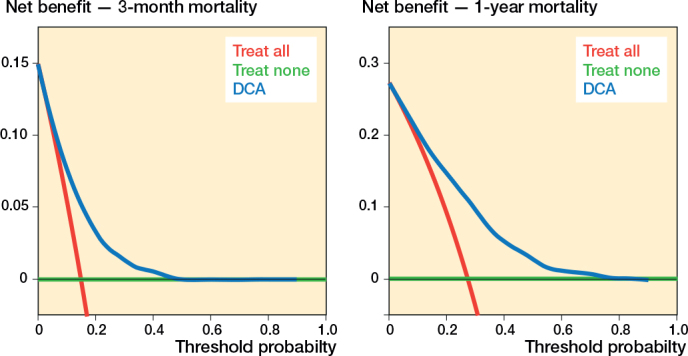
Decision curve analysis for external validation of recalibrated XGB model for 3-months and 1-year mortality.

Table 3 presents observed event rates and model performance metrics, including average predicted risk, calibration slope, intercept, and AUC, at 1, 3, 6, and 12 months, before and after isotonic recalibration. ROC curves for 3- and 12-month mortality are shown in Figure 2, flexible calibration curves for 3- and 12-month mortality before recalibration in Figure 3, and the recalibrated XGB models and DCA curves for the same timepoints in Figures 4 and 5.

**Table 3:** Summarized results of model performance for different timepoints before and after calibration

Models	Average predicted risk	Slope	Intercept	AUC (CI)
1 month – overall event rate in test set 0.08				
Uncalibrated models				
XGB	0.12	0.80	–0.80	0.72 (0.70–0.74)
GLM	0.12	0.06	–2.17	0.70 (0.67–0.72)
RF	0.10	0.55	–1.02	0.74 (0.72–0.76)
After calibration **^a^**				
XGB	0.07	0.88	0.003	0.72 (0.70–0.74)
3 months – overall event rate in test set 0.15				
Uncalibrated models				
XGB	0.22	0.95	-0.60	0.74 (0.72–0.76)
GLM	0.22	0.21	-1.36	0.71 (0.69–0.73)
RF	0.06	0.40	-0.24	0.73 (0.72–0.75)
After calibration **^a^**				
XGB	0.12	0.89	0.07	0.74 (0.72–0.76)
6 months – overall event rate in test set 0.20				
Uncalibrated models				
XGB	0.30	0.97	-0.62	0.75 (0.73–0.76)
GLM	0.28	0.19	-1.10	0.73 (0.71–0.74)
RF	0.09	0.64	0.34	0.75 (0.73–0.77)
After calibration **^a^**				
XGB	0.17	0.90	0.03	0.75 (0.73–0.76)
1 year – overall event rate in test set 0.27				
Uncalibrated models				
XGB	0.38	1.09	-0.57	0.77 (0.75–0.78)
GLM	0.35	0.14	-0.81	0.74 (0.72–0.75)
RF	0.18	0.67	0.21	0.75 (0.74–0.76)
After calibration **^a^**				
XGB	0.24	0.94	0.16	0.77 (0.75–0.78)

a calibration with isotonic regression.

## Discussion

The aim of our study was to externally validate our previously published ML models for prediction of mortality after hip fracture in an independent Swedish cohort. The models showed consistent performance across timepoints, with XGB achieving AUCs of 0.72–0.77 for 1-, 3-, 6-, and 12-month mortality. Because the overall event rate differed between the development and validation cohorts, recalibration was performed, resulting in good calibration at large and acceptable intercept, slope, and DCA. Although the cohorts differed in sex, hemoglobin, ASA score, and cardiac medication, this reflects case-mix shifts in prevalence rather than changes in predictor effects. Because the model predicts individual risk, stable discrimination and calibration following event-rate adjustment suggest limited influence of these differences on performance.

For practical use, it was unnecessary to provide all 4 timepoints in the online tool so the 3- and 12-month models were therefore prioritized. A meta-analysis of over 700,000 individuals found that the highest relative risk for mortality after a hip fracture was during the first 3 months [14], supporting selection of the 3-month model over the 1-month model, which also showed poorer discrimination. The 12-month model was included to provide a longer-term estimate commonly reported in the literature. There is a large discrepancy in the literature between the amount of work published for development and for external validation of prediction models in this population. Our evaluation followed the “ABCD” structure proposed by Steyerberg and Vergouwe [9] and considered calibration at large, calibration slope and intercept, discrimination, and decision curve analysis.

Much of the previous literature for external validation was done with a different methodology than in the present study, but as demonstrated by van Calster et al. [11] there is a need to consider calibration when evaluating ML models, as reporting only AUC can be misleading. This is also evident by comparing the results and calibration curves in the present study before and after recalibration.

In the same article it is recommended to avoid the Hosmer–Lemeshow test as a measure of calibration as this does not consider the extent or type of the miscalibration and as such does not evaluate calibration sufficiently.

A meta-analysis compared the performance of ML models with previously published models for estimating mortality in this population and found that, overall, the ML methods was more accurate [3]. One of the most extensively researched previous models not employing ML techniques is the Nottingham Hip Fracture Score (NHFS) and the pooled AUC for external validation of that model was 0.70 (0.68–0.72) in the meta-analysis. External validation has also been done for the NHFS score for a Swedish population with similar results; in a study of 998 patients the AUC for 30-day mortality was 0.67 (0.59–0.74) [15].

None of the 14 articles that was pooled used a similar evaluation of calibration to that in our study. Instead, the Hosmer–Lemeshow test or observed/expected ratios was reported, and in some studies calibration was not discussed, making comparisons with our study difficult.

In a different article on a similar topic, a systematic review and meta-analysis was performed on articles concerning the use of Artificial Intelligence (AI) for hip fracture detection on radiographs and outcome prediction [2]. Only a minimal advantage of using ML based methods for prediction of outcomes in comparison with regression type statistics was found, but that reporting heterogeneity makes comparisons difficult was also highlighted. Also of note was that none of the 39 studies evaluated in that article had performed external validation.

Lei et al. [1] developed and externally validated ML models for predicting in-hospital mortality after hip fracture in critical care patients using gradient boosting, decision tree, RF, and XGB models. External validation included 165 patients, of whom 18 died. The XGB model performed best, but the confidence interval was very wide (AUC 0.72, CI 0.57–0.86), and the calibration slope and intercept were worse than our figures both before and after recalibration. Flexible calibration curves were presented but difficult to interpret due to the small sample size. The small, highly selected population limits generalizability to routine clinical care. It is unclear whether the performance reflects that the smaller sample size gives unrepresentative results or if the actual precision of the model is worse.

Another model for prediction of in-hospital mortality in this patient group is the so-called U-Hip, which has been externally validated by Schuijt et al. [16]. In line with our views, they point out the lack of externally validated mortality models in this population. In-hospital mortality is a frequently used timeframe in geriatric traumatology, even though discharge policies and the resulting length of stay may be different between centers and it can be argued that any type of increased mortality is of equal importance. However, in-hospital mortality might be of interest to identify patients who could benefit from changes in care while admitted.

The study is based on 25,052 patients from over 150 different hospitals and had good calibration and an AUC of 0.74 (CI 0.72–0.76), so results were similar to our results for 1-month mortality, but it is unclear what the timeframe was in that study.

Given the diversity in populations, treatment protocols, mortality rates, and outcome reporting across studies, it is unlikely that a single model will be applicable to all hip fracture populations without recalibration or updating during external validation before clinical use. Well-executed studies can nevertheless serve as benchmarks when updating or developing new models, and ML may support more accurate and personalized care in hip fracture management.

### Limitations

Missing data was present in the predictors but assumed to be reliably imputed and of course difficult to avoid in clinical data.

The data in the study was not specifically collected for this purpose and the parameter ”New Mobility Score” had to be created from other parameters in the external validation set.

The event rate in the external validation was lower than in the development, necessitating recalibration of the model under study.

The data was from 2 geographically different hospitals but under the same general management, so presumably more similar than 2 completely different hospitals would have been.

Comparative analyses against existing prognostic models, such as the Nottingham Hip Fracture Score or other ML models, would have provided additional insights into the incremental value of our model in mortality prediction, but the necessary predictors for this assessment were not available to us.

### Conclusions

External validation of the previously published models confirmed the original findings, with the XGB models again demonstrating the best overall performance. Recalibration addressed cohort differences in mortality rates and resulted in well-aligned predictions. The models showed acceptable discrimination, were well calibrated after recalibration for differing event rates between development and validation cohorts, and demonstrated net benefit on decision curve analysis across a wide range of thresholds. This supports suitability for clinical use, and the recalibrated models may help identify patient subgroups at different levels of risk to prioritize care and guide research. An updated online calculator including the recalibrated 3- and 12-month models is available (https://hipfx.shinyapps.io/hipfxswe/).

## Notes

MM prepared the first draft of the paper and did the statistical analysis of the data. All authors were involved in the design of the study and interpretation of the results. All authors revised the paper critically for intellectual content and approved the final version.

Handling co-editors: Taco Gosens and Robin Christensen

*Acta* thanks Anne Marie Fenstad and Bart G Pijls for help with peer review of this manuscript.
